# Medicinal value of asiaticoside for Alzheimer’s disease as assessed using single-molecule-detection fluorescence correlation spectroscopy, laser-scanning microscopy, transmission electron microscopy, and *in silico* docking

**DOI:** 10.1186/s12906-015-0620-9

**Published:** 2015-04-14

**Authors:** Shahdat Hossain, Michio Hashimoto, Masanori Katakura, Abdullah Al Mamun, Osamu Shido

**Affiliations:** Department of Environmental Physiology, Shimane University Faculty of Medicine, Izumo, 693-8501 Japan; Department of Biochemistry and Molecular Biology, Jahangirnagar University, Savar, Dhaka Bangladesh

**Keywords:** Asiaticoside, Amyloid fibrillation, Fluorescence correlation spectroscopy, Neurotoxicity, Molecular Docking

## Abstract

**Background:**

Identifying agents that inhibit amyloid beta peptide (Aβ) aggregation is the ultimate goal for slowing Alzheimer’s disease (AD) progression. This study investigated whether the glycoside asiaticoside inhibits Aβ_1–42_ fibrillation *in vitro*.

**Methods:**

Fluorescence correlation spectroscopy (FCS), evaluating the Brownian diffusion times of moving particles in a small confocal volume at the single-molecule level, was used. If asiaticoside inhibits early Aβ_1–42_ fibrillation steps, more Aβs would remain free and rapidly diffuse in the confocal volume. In contrast, “weaker or no inhibition” permits a greater number of Aβs to polymerize into oligomers, leading to fibers and gives rise to slow diffusion times in the solution. Trace amounts of 5-carboxytetramethylrhodamine (TAMRA)-labeled Aβ_1–42_ in the presence of excess unlabeled Aβ_1–42_ (10 μM) was used as a fluorescent probe. Steady-state and kinetic-Thioflavin T (ThT) fluorospectroscopy, laser-scanning fluorescence microscopy (LSM), and transmission electron microscopy (TEM) were also used to monitor fibrillation. Binding of asiaticoside with Aβ_1–42_ at the atomic level was computationally examined using the Molegro Virtual Docker and PatchDock.

**Results:**

With 1 h of incubation time for aggregation, FCS data analysis revealed that the diffusion time of TAMRA-Aβ_1–42_ was 208 ± 4 μs, which decreased to 164 ± 8.0 μs in the presence of asiaticoside, clearly indicating that asiaticoside inhibited the early stages Aβ_1–42_ of fibrillation, leaving more free Aβs in the solution and permitting their rapid diffusion in the confocal volume. The inhibitory effects were also evidenced by reduced fiber formation as assessed by steady-state and kinetic ThT fluorospectroscopy, LSM, and TEM. Asiaticoside elongated the lag phase of Aβ_1–42_ fibrillation, indicating the formation of smaller amyloid species were impaired in the presence of asiaticoside. Molecular docking revealed that asiaticoside binds with amyloid intra- and inter-molecular amino acid residues, which are responsible for β-sheet formation and longitudinal extension of fibrils.

**Conclusion:**

Finally, asiaticoside prevents amyloidogenesis that precedes neurodegeneration in patients with Alzheimer’s disease.

## Background

Alzheimer’s disease (AD) is pathologically characterized by the fibrillar deposition of amyloid beta peptides (Aβ) in the brain [1]. Aβ_1–40_ prevails in the cerebrospinal fluid of patients with AD [2]. However, Aβ_1–42_ deposition prevails in the brains of patients with AD [3], thereby implicating its involvement in the initiation of fibrillation. However, the mechanisms of Aβ fibrillation remain elusive. Several key events are considered to be crucial, including the mechanism by which Aβ_1–42_ abandons its random coil-alpha helix conformation and adapts a β-sheet conformation, leading to oligomerization and finally matured fiber formation. These α-to-β conformational transitions are crucial for understanding the mechanisms of fiber formation and inhibition by agents capable of inhibiting the fibrillation process.

Asiaticoside, a highly polyphenolic compound, is one of the major triterpene glycosides in *Centella asiatica* (CA). CA is an extremely important medicinal herb used in Java and other Indonesian islands, China [4], and other Asian countries [5], and is also becoming popular in Western countries [6]. Asiaticoside retains inherent properties to combat oxidative species, which are frequently observed in patients with AD-associated memory impairments [7,8]. CA increases intelligence and memory [9]. Asiaticoside also has been reported to protect against Aβ-induced neurotoxicity [10]. More importantly, asiaticoside has been patented as a dementia-treating agent and cognitive enhancer [11]. However, the mechanisms of action of asiaticoside have remained largely unknown.

In this study, we primarily used fluorescence correlation spectroscopy (FCS) to examine whether asiaticoside inhibits Aβ_1–42_ fibrillation and its mechanism. FCS is a new method that can detect molecular motion at the nanomolar level in small sample volumes. This technique has recently attracted interest for investigating the molecular interactions of proteins [12,13]. FCS allows real-time monitoring of protein–protein interactions in a reaction solution without separation of the free and bound forms [14,15]. For example, FCS can be successfully used in an aggregating system using trace amounts of 5-carboxytetramethylrhodamine (TAMRA)-labeled Aβ_1–42_ in the presence of a large excess of unlabeled Aβ_1–42_ in a solution [16]. During aggregation, the fluorescent species will remain constant (because of the large excess of unlabeled molecules), and the diffusion time will gradually increase. Fluctuations in the fluorescence signal in a detection volume of approximately 1 fl (femtoliter) are analyzed using an autocorrelation function, revealing information about the diffusion properties of the fluorescent complexes; larger average complex sizes are associated with longer diffusion times. Changes in the average diffusion time reflect changes in the complex size and/or the ratio of free fluorescently labeled molecules in the complexes.

In addition, steady-state and kinetic thioflavin T (ThT) fluorospectroscopy, transmission electron microscopy (TEM), and laser-scanning fluorescence microscopy (LSM) were used to elucidate the mechanism of asiaticoside-induced inhibition of Aβ_1–42_ fibrillation. In the field of molecular modeling, docking is a method that predicts the preferred orientation of one molecule to a second when bound to each other as a stable complex [17]. At present, the use of computers to predict the binding of small molecules to known target protein structures has been an important component in the drug discovery process [18,19]. However, there is no conclusive report regarding whether the asiaticoside docks onto Aβ_1–42_, and if so, the amino acid specificity with which it binds as ligand to inhibit amyloid aggregation is unclear. We, therefore, investigated whether asiaticoside binds with amyloidogenic hot spots, i.e., the amino acid residues involved in β-aggregation, which may further support the use of asiaticoside as an amyloidogenesis-inhibitory agent.

## Methods

### Materials

Aβ_1–42_ (human, 1–42) was purchased from the Peptide Institute (Osaka, Japan). Asiaticoside was purchased from Sigma-Aldrich. The reference dye 5-carboxytetramethylrhodamine (TAMRA) was purchased from Olympus America Inc, whereas TAMRA-Aβ_1–42_ was obtained from ANASPEC Inc. CA. Other chemicals were of analytical grade. Uranyl acetate was obtained from BDH. All experiments were carried out with the approval of an appropriate ethics committee of Shimane University compiled from the Guidelines for Animal Experimentation of the Japanese Association for Laboratory Animal Science.

### Preparation of asiaticoside, TAMRA-Aβ_1–42_, and unlabeled Aβ_1–42_

Asiaticoside was dissolved in ethanol, diluted, N_2_-dried to remove ethanol, and then mixed with assembly buffer to have final concentrations of 5, 10 and 20 μM. TAMRA-Aβ_1–42_ and unlabeled Aβ_1–42_ were dissolved in hexafluoroisopropanol (HFIP), aliquoted, and stored at −80°C until use. HFIP was also blown with N_2_ prior to the use in fibrillation assay.

### Fluorescence Correlation Spectroscopy (FCS)

#### Theory

**Figure 1:**
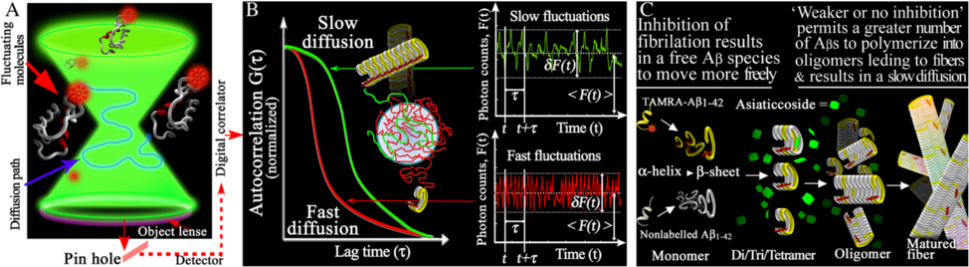
Schema of fluorescence correlation spectroscopy (FCS) measurement. **(A)**. Confocal volume of FCS measurement. A pinhole provides an axial confinement to results in a tiny (less than femtoliter) detection volume (confocal volume). The fluorescence from the confocal volume is detected and processed using a digital correlator. **(B)**. Principle of autocorrelation analysis of FCS measurements. δF(t), the fluorescence intensity fluctuations; <F(t)>, time average (< >) fluorescence intensity; G(τ), the auto-correlation curve is constructed from δF(t). **(C)**. Mechanism of asiaticoside-induced inhibition at early steps of Aβ_1–42_ fibrillation.

In an FCS experiment, fluctuations of the fluorescence δF(t) around the average fluorescence <F(t)> are measured, yielding information on molecular processes or motions. The fluctuations of the fluorescence signal, δF(t), stem from changes in either the number of fluorescent particles or the fluorescence quantum yield of the particles in the open probe volume, which is defined by the confocal volume of a tightly focused laser beam. To analyze these fluctuations, the autocorrelation function G(τ) of the fluorescence intensity is calculated using the following equation:

$$ \mathrm{G}\left(\uptau \right)=\frac{\left\langle \updelta \mathrm{F}\left(\mathrm{t}\right)\ \updelta \mathrm{F}\left(\mathrm{t}+\uptau \right)\right\rangle }{{\left\langle \mathrm{F}\left(\mathrm{t}\right)\right\rangle}^2} $$

where τ is the correlation time. F(t) signifies the detected fluorescence intensity, where δF(t) is the variable, fluctuating part and <F(t)> denotes the mean. The angular brackets indicate a time average, <δF(t) δF(t + τ) **>** is the average product of a fluctuations amplitude at time t and a later time (τ), (**t** + τ). F denotes the fluorescence signal as a function of time (see Figure 1).

FCS measurements were performed on a Fluoro Point Light system (Olympus, Tokyo, Japan) at room temperature using the onboard 543-nm helium–neon laser at a power of 100 μW for excitation, as previously described [20]. TAMRA-Aβ_1–42_/unlabeled Aβ_1–42_ dissolved in HFIP was blown with N_2_ gas and re-dissolved in the assembly buffer with or without asiaticoside. Free TAMRA (rhodamine) was used as a reference dye. The final concentrations of TAMRA-Aβ_1–42_ and unlabeled-Aβ_1–42_ were 5 nM and 10 μM, respectively. The measurements were performed in a sample volume of 50 μl in the 384-well glass-bottomed microplate. The samples were sequentially and automatically loaded into the device. FCS measurements were conducted at 0 and 1 h with a data acquisition time of 10 s per measurement, and measurements were repeated five times per sample.

### Steady-state Aβ_1–42_ fibrillation analysis using ThT fluorospectroscopy

Aβ_1–42_ fibrillation was induced as previously described [8,21]. After blowing HFIP from the Aβ_1–42_ stock aliquot, the dried peptide (50 μM) was suspended in desired volume of assembly buffer (100 μl of 50-mM Tris–HCl buffer, pH 7.4, containing 100 mM NaCl and 0.01% sodium azide) with or without asiaticoside (final concentrations of asiaticoside were 0, 5, 10, and 20 μM). The reaction mixture was taken in oil-free PCR tubes (Takara Shuzo, Otsu, Japan) flushed with of N_2_ gas to obviate any effect of atmospheric oxygen, and incubated at 37°C for 24 h into a DNA thermal cycler (PJ480; Perkin Elmer Cetus, Emeryville, CA). After 24 h, the incubation was stopped by placing the tubes on ice and 40-μl aliquots from each tube were mixed with 210 μl of 5-μM ThT in 50 mM glycine–NaOH buffer (pH 8.5) and subjected to fluorospectroscopy (Hitachi F-2500 fluorescence spectrophotometer) at excitation and emission wavelengths of 448 and 487 nm, respectively.

### Effects of asiaticoside on the kinetics of Aβ_1–42_ fibrillation

The effect of asiaticoside on the kinetics of fibrillation was evaluated by using 10 μM of Aβ_1–42_. Briefly, the reaction mixture containing 1.0 ml of 50 mM Tris–HCl buffer, pH 7.4, 100 mM NaCl, 0.01% sodium azide, and the desired amount of Aβ_1–42_ with or without asiaticoside (20 μM) was taken into Eppendorf tubes and incubated at 37°C. At desired time intervals, a 40-μl of the peptide mixture was gently removed and added to 210 μl of 5 μM ThT in 50 mM glycine–NaOH buffer (pH 8.5) for fluorescence assay.

### Aβ_1–42_ fibrillation analysis using Laser Scanning Microscopy (LSM)

A 2.5-μl aliquot of the fibrillated Aβ_1–42_ peptide (50 μM) sample from the ThT-Aβ_1–42_ assay with or without asiaticoside (20 μM) was diluted 2-fold with 5 μM ThT in 50 mM glycine–NaOH buffer (pH 8.5), transferred onto slides, and photographed using the confocal laser microscope system (CLSM FV300; Olympus). The fibrillation of 5 nM TAMRA-Aβ_1–42_ + 20 μM unlabeled Aβ_1–42_ with or without 20 μM asiaticoside was also conducted. Aggregates of TAMRA-Aβ_1–42_ were directly visualized under the microscope at an excitation wavelength of 542 nm.

### Aβ_1–42_ fibrillation analysis using TEM

Fibrillation of Aβ_1–42_ (50 μM) was induced as described for ThT fluorospectroscopy. A 4-μl aliquot was used for electron microscopy. In brief, a droplet of the reaction mixture was spread onto carbon-coated grids, negatively stained with 1% uranyl acetate (pH 7.0), and examined under a Hitachi H-7000 electron microscope with an acceleration voltage of 75 kV.

### Docking study: Preparation of docking materials (ligand and receptor)

**Figure 2:**
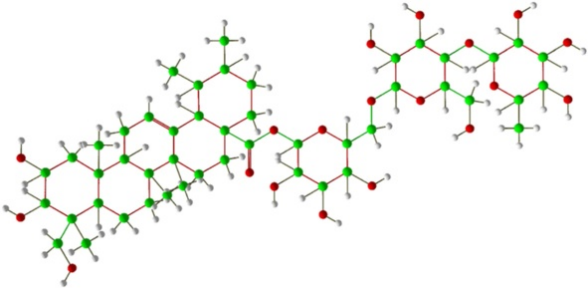
All-atoms molecular structure of asiaticoside (PubChem: CID 108062).

The canonical SMILES string of asiaticoside (PubChem: CID 108062; Figure 2) was submitted to Marvin 5.7.0 [22] to generate the 3D structure of this ligand molecule. The 3D structure of asiaticoside was subsequently energy-minimized and converted to the Protein Data Bank (PDB) file format using the Molegro Virtual Docker (MVD) [23]. Aβ_1–42_ (PDB ID: 2BEG) was downloaded from the Protein Data Bank as a receptor for asiaticoside docking. 2BEG is a 3D NMR structure of Aβ_1–42_, consisting of a homopentamer (A, B, C, D, and E). Each monomer of the 2BEG pentamer comprises 10 coordinate models [24].

### Dimer formation

At least two amyloid molecules are required to achieve the repeating structure of a protofilament fibril; therefore, the coordinates of model 1 of A monomer (A1) and model 1 of B monomer (B1) were split from the composite 2BEG PDB file by MVD. Subsequently, the monomer (A1)–monomer (B1) dimer (A1-B1) was generated by feeding the A1 and B1 monomers to RosettaDock [25], which decoys 1000 independent simulations and gives the A1–B1 dimer on the basis of energy minimization.

### Analysis of the unstructured and aggregation-prone regions of Aβ _1–42_

The FASTA format of the primary amino acid sequence of Aβ_1–42_ (>seq DAEFRHDSGYEVHHQKLVFFAEDVGSNKGAIIGLMVGGVVIA) was tested by ANCHOR [26] to elucidate the intrinsically unstructured region of this peptide. In addition, we used three state-of-the-art sequence-based computational methods, namely **FoldAmyloid** [27], AGGRESCAN [28], and ProA [29], to predict the amyloidogenic regions and aggregation-prone amino acid residues from Aβ_1–42_ peptide chains.

### Computational analysis of binding sites of Aβ _1–42_ monomers and dimers

The binding sites or pockets of the A1-monomer and A1–B1 dimer were determined by GHECOM, which detects grid-based pockets/cavities on the surface of the protein [30].The program produces a graph of residue-based pocketness. The presence of binding sites was also cross-checked using Q-SiteFinder [31], which uses the interaction energy between the protein and a simple van der Waals probe to locate energetically favorable binding sites.

### Intersurface interaction site analysis of the A1–B1 dimer

Protein–protein, i.e., monomer-monomer A1-B1 dimer, intersurface interaction sites were analyzed by feeding the A1–B1 dimer to the cons-PPISP server [32], which predicts the residues that likely form the binding site for neighbor proteins. To further validate the presence of intersurface hot points on the dimer interface binding contacts, the dimer was fed to hot point prediction servers, including KFC2 [33,34].

### Docking simulation of asiaticoside onto Aβ_1–42_ monomer (A1) and dimer (A1–B1)

The molecular docking simulations were performed using the Molegro Virtual Docker (MVD) [23] and PatchDock [35].

### Molegro Virtual Docker

MVD is an automated docking software program with fast processing that automatically adds the missing hydrogen atoms of the ligand and receptor molecules, if any. The software also has a module to create a surface over the receptor molecule and identify potential binding sites for its activity. The program gives 10 conformational positions or so-called poses for the ligand and returns the five best poses with MoleDockScore (equivalent to energy of binding/docking energy) and other thermodynamically calculated values. The MoleDockScore is an anonymous value by which one can suggest the best-docked ligand with its conformation. MVD also presents hydrogen bond information together with other thermodynamic values that suggest the formation of stable complexes between ligands and receptor molecules. MVD performs flexible ligand docking with optimization of the ligand geometry during docking, thus indicating bond angles, bond lengths and torsional angles of the ligand are modified at the stages of receptor-ligand complex generation.

### PatchDock

PatchDock is a shape complementarity/geometry-based molecular docking algorithm. It is aimed at finding docking transformations that yield good molecular shape complementarity. Such transformations induce both wide interface areas and small amounts of steric clashes. A wide interface is ensured to include several matched local features of the docked molecules that have complementary characteristics. The ouput of PatchDock is a list of candidate complexes between receptor and ligand molecule. The list is sorted according to geometric shape complementarity score, approximate interface area of the receptor-ligand complex; atomic contact energy (ACE) between ligand and receptor, and 3D transformation. Finally, the server provides an option to download the ligand-receptor complexes in the PDB format.

### Statistical analysis

Results were expressed as the mean ± S.E. (standard error of mean). For intergroup differences, the data were analyzed by unpaired student’s *t*-test and one-way analysis of variance (ANOVA: for more than 2 groups). ANOVA was followed by Fisher’s Protected Least Significant Difference (PLSD) for post hoc comparisons. Kinetic data of nucleation-dependent fibrillation were subjected to non-linear variable-slope sigmoidal equation. The statistical program used was StatView® 4.01 (MindVision Software, Abacus Concepts, Inc., Berkeley, CA, USA) and GRAPHPAD PRISM (version 4.00; GraphPad Software Inc., San Diego, CA, USA). A level of P < 0.05 was considered statistically significant.

## Results

### Effect of asiaticoside on the diffusion time of Aβ_1–42_

**Figure 3:**
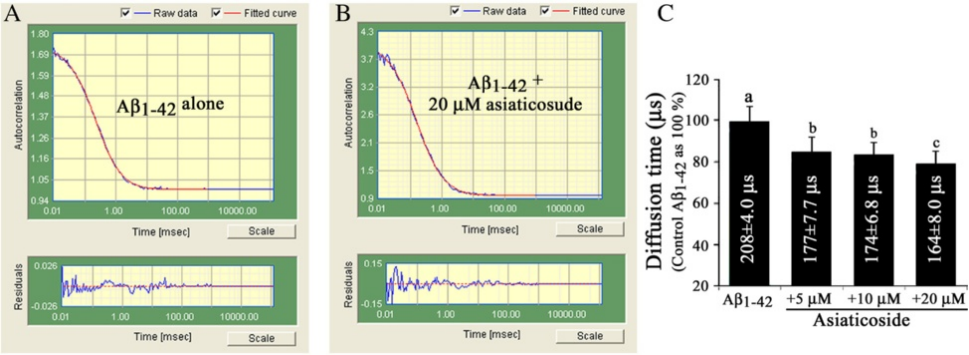
Autocorrelation curves G(τ) of fluorescence fluctuations. Representative autocorrelation curves G(τ) of fluorescence fluctuations δF(t) of TAMRA-Aβ_1–42_ (5 nM) mixed with excess (20 μM) unlabeled Aβ_1–42_ without (**A**, control) or with 20 μM asiaticoside **(B)**. The blue lines represent the raw data, whereas the red lines represent fitted data. The curve fit was good as seen in the residuals, which are the differences between the data and the fitted curve. The residuals of a good fit fluctuate randomly and tightly about zero. **(C)**. Diffusion times of TAMRA-Aβ_1–42_ during Aβ_1–42_ fibrillation in the absence (Aβ_1–42_ alone, control) and presence of different concentrations of asiaticoside (5–20 μM). Results are presented as the mean ± SE of quadruplicate determinations (n = 5).

The diffusion time of the reference TAMRA dye was 100 ± 5 μs and that of the TAMRA-Aβ_1–42_ alone was 150 ± 5 μs. The autocorrelation function of TAMRA-Aβ_1–42_ in the (TAMRA-Aβ_1–42_ + unlabeled Aβ_1–42_) sample was best fitted with a one-component analysis model, which resulted in a diffusion time of 208 ± 4 μs (Figure 3A). However, the diffusion time of TAMRA-Aβ_1–42_ in the (TAMRA-Aβ_1–42_ + unlabeled Aβ_1–42_ + 20 μM of asiaticoside) samples was reduced to 164 ± 8.0 μs (Figure 3B).

### Effect of asiaticoside on steady-state fibrillation of Aβ_1–42_

**Figure 4:**
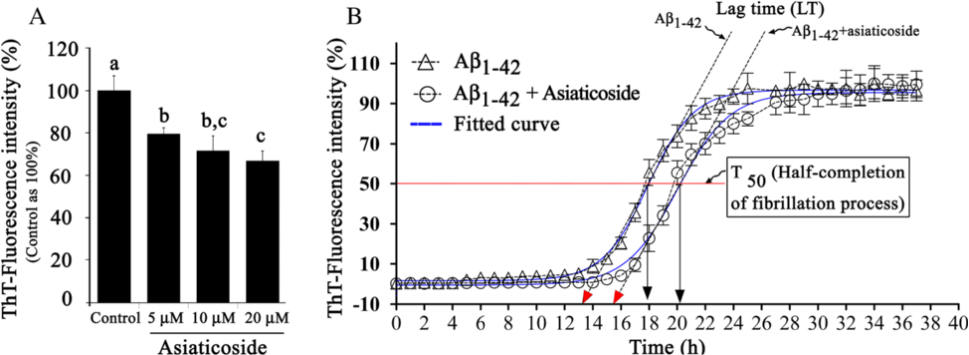
Effects of asiaticoside on the fibrillation of Aβ_1–42_. **(A)**. Steady-state fibrillation. ThT, thioflavin T; Aβ_1–42,_ 50 μM. Bar with different letters indicates significant difference (P < 0.05). Mean ± SE, n = 3. **(B)**. Kinetics of fibrillation. Aβ_1–42,_ 10 μM; Bar, mean ± SE, n = 3.

The fibrillation of Aβ_1–42_ only, as measured by the steady-state ThT fluorescence intensity, was set as 100% (control). Asiaticoside at 5, 10, and 20 μM significantly (0 < 0.05) decreased fibril formation by 20, 29, and 33%, respectively, compared with that of the control (Figure 4A). After examining the inhibitory effect of the three concentrations of asiaticoside on Aβ_1–42_ fibrillogenesis, we also studied the effect of 20 μM asiaticoside on the kinetics of Aβ_1–42_ fibrillation.

### Effects of asiaticoside on the kinetics of Aβ_1–42_ fibrillation

Fibrillation kinetics of Aβ_1–42_ at 50 μM of concentration (the concentration used for steady-state ThT assay) displayed a very short lag phase (<10 min) at our experimental condition (data not shown)). This led us to assess the effect of asiaticoside on the kinetics of Aβ_1–42_ fibrillation at 10 μM of concentration. At this low concentration, fibrillation appeared to be highly cooperative, reminiscent of first order phase transitions and the fibrillation kinetics exhibited characteristics of a typical nucleation and growth process (Figure 4B). The time course of fibrillation included a lag phase followed by a rapid exponential growth (elongation) of fibrils. Experimental data were fitted well by such a variable-slope sigmoidal equation curve: [Y = Bottom + (Top - Bottom)/(1 + 10^((LogT_50_-X)*HillSlope)); where, Bottom is the Y value at the bottom plateau, Top is the Y value at the top plateau, LogT_50_ is the X value when the ThT fluorescence is halfway between Bottom and Top, HillSlope describes the steepness of the curve]. Lag time (LT) was defined as the time at which the tangent at the point of the maximum fibrillation rate intersects the abscissa. At 95% confidence interval, T_50_ (half-life) was increased by asiaticoside (T_50_: 10 μM of Aβ_1–42_ vs. 10 μM of Aβ_1–42_ + asiaticoside = 17.9 ± 0.07 vs. 20.5 ± 0.13 h; with a corresponding LT of 13.4 and 15.4 h, respectively. Asiaticoside lengthened the time both at the lag phase and during the growth of fibrillation for 2 - 3 h.

### Laser Scanning Microscopy (LSM)

**Figure 5:**
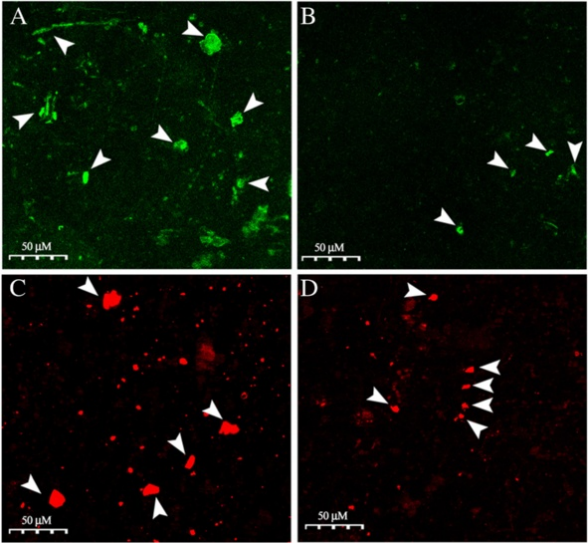
Visualization of amyloid aggregates by laser-scanning microscopy (LSM). Images were taken after overnight of aggregation at 37°C with a concentration of 50 μM unlabeled Aβ_1–42_ **(A)** and 5 nM TAMRA-Aβ_1–42_ + 20 μM unlabeled Aβ_1–42_ **(C)**. Aggregates of (50 μM unlabeled) Aβ_1–42_ displayed characteristic green fluorescence after their binding with ThT. TAMRA-Aβ_1–42_ itself exhibited orange fluorescence. Asiaticoside-incubated samples had a lower number and smaller green (ThT) **(B)** and orange (TAMRA-Aβ_1–42_) **(D)** fluorescent areas than those of the controls, suggesting that Aβ_1–42_ fibrillation was inhibited by asiaticoside.

The fibrillations of unlabeled Aβ_1–42_ and asiaticoside + unlabeled Aβ_1–42_ samples also were examined by ThT laser scanning fluoromicroscopy (LSM) (Figure 5A, B), which gave green fluorescence. The fibrillations of TAMRA-Aβ_1–42_ + unlabeled Aβ_1–42_ and asiaticoside + TAMRA-Aβ_1–42_ + unlabeled Aβ_1–42_ samples were also examined by laser-scanning microscopy (LSM). TAMRA-Aβ_1–42_ evoked characteristic orange fluorescence (Figure 5C, D). The results disclosed the precipitation of large aggregates on the glass slides. In addition, the images were processed by ImageJ to calculate the areas of the green or red fluorescence Aβ-aggregates. Area < 5.0 μM^2^ was not included in the image processing. The Aβ_1–42_ + asiaticcoside samples had a lower number of green-fluorescent areas than did the Aβ_1–42_ untreated controls. Total green fluorescent areas were 768.7 ± 9.9 and 92.1 ± 3.4 μM^2^, respectively in the controls and asiaticcoside-treated samples (Figure 5A, B), suggesting that the aggregations of Aβs were inhibited by asiaticcoside. The red spots (Figure 5C, D) are amyloid deposits of labeled amyloid Aβ_1–42_ (TAMRA-Aβ_1–42_). Again, the number of red spot areas was significantly lesser in the presence of asiaticoside, indicating asiaticoside inhibited the fibrillation and consequently the amyloid deposits. Total areas of red-fluorescence areas were, respectively, 2513 ± 9.4 and 481 ± 18.7 μM^2^ for the control and asiaticoside-treated samples.

### Effect of asiaticoside on Aβ_1–42_ morphology

**Figure 6:**
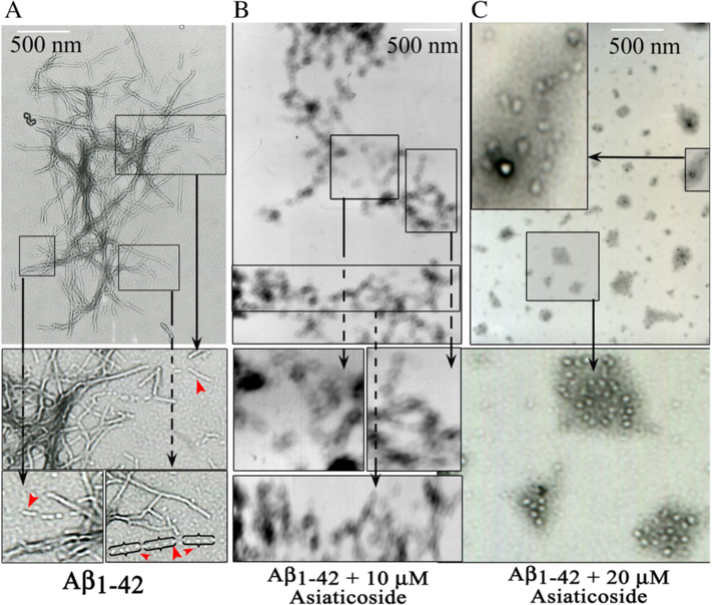
Representative transmission electron microscopic (TEM) views of the effects of asiaticoside on fibrillation. **A**: Control (Aβ_1–42_ alone), Insets of A show complete fibers, protofibrills, and oligomeric globular amyloid species appeared to arrange in lines (red arrow) to form long fibers; **B**: Aβ_1–42_ + 10 μM asiaticoside; and **C**: Aβ_1–42_ + 20 μM asiaticoside. The fibers (**B**, **C**) became unstructured, diffused, and irregular, sometimes they appeared as globular structures (insets of **B** and **C**). The globular structures appeared more dispersed at 20 μM of concentrations of asiaticoside than those at 10 μM. These results indicate that asiaticoside clearly inhibited Aβ_1–42_ fibrillation.

The control samples (Aβ_1–42_ alone) contained abundantly aggregated amyloid fibrils in the assembly buffer, whereas the Aβ_1–42_ + asiaticoside samples contained only very small amounts of aggregates. The former fibrils exhibited a typical filamentous and branching morphology (Figure 6A and its inset). The lengths of the fibers differed from grid to grid, and the fibers were nearly inconspicuous because of the presence of extensive branching. The fibers of the Aβ_1–42_ + asiaticoside samples (Figure 6B) displayed spaced, beaded and spheroidal structures, leading to necklace-like diffused proto-fibrillar filaments. The protofibrillar structures were found to align along the long axis of the fibers as if, they were ‘on pathway’ to complete the long fibers (arrow head). In the presence of 20 μM asiaticoside, these fibers exhibited as more diffused, oligomeric and cluster-like structures.

### Salient features of A1 monomers and A1–B1 dimers

**Figure 7:**
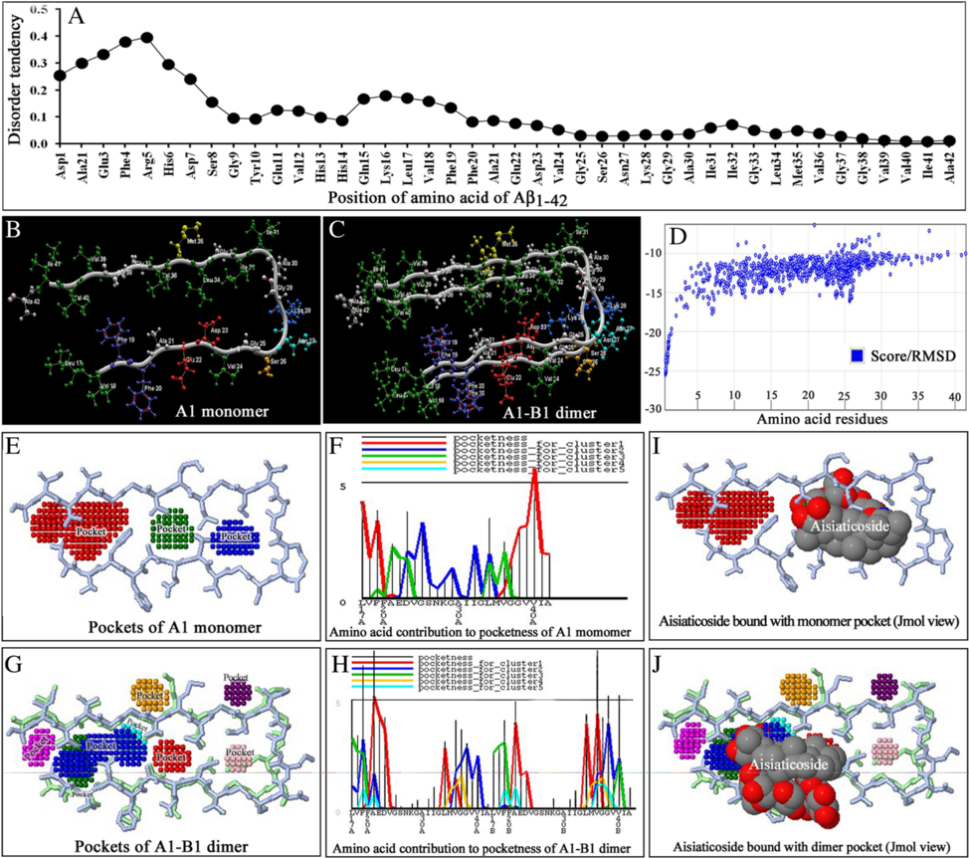
Features of the primary amino acid sequence of Aβ_1–42_. **A**: Prediction of the intrinsically unstructured amino acid region of Aβ_1–42_ by the ANCHOR server. ANCHOR identifies segments in a generally disordered region that cannot form sufficient numbers of favorable intrachain interactions. The amino acid residues from Asp1–Val18 exhibited unstructured characteristics. **B**: A1 monomer model of the Protein Data Bank (PDB) file of Aβ_1–42_ (2BEG), split by the Molegro Virtual Docker (MVD). **C**: Dimer (A-B1) of the A1 and B1 monomer of 2BEG. The dimer was formed by feeding the A1 and B1 monomers to RosettaDock. **D**: Validation graph of dimer (A1-B1) formation by RosettaDock. A hallmark of a successful dimer run is an energetic “funnel” of low-energy structures clustered around a single position. **E,** **G**: Grid-based pocketness cluster of the A1 monomer **(E)** and dimer (A1-B1) **(G)** by GHECOM. **F,** **H**: Contributions of amino acids of the A1 monomer **(F)** and A1–B1 dimer **(H)** to cluster pocketness. The line shows the value of pocketness [%] for each residue. A residue in a deeper and larger pocket has a larger value of pocketness. The color of the pocketness bar indicates the cluster number of pockets. **I,** **J**: The views were generated in Jmol viewer after the docking of asiaticoside onto the (A1) monomer and dimer (A1–B1) to demonstrate that the ligand (asiaticoside) truly bound with the pockets and/or binding sites.

The ANCHOR server (http://iupred.enzim.hu/) identified amino acids 1–18 that remained in a generally disordered region. These amino acids do not adopt a stable structure and cannot engage in sufficient numbers of favorable intrachain interactions (Figure 7A). The A1 monomer split from 2BEG is shown in Figure 7B, while the A1-B1 dimer generated by RosettaDock is shown in Figure 7C. The RosettaDock server gave the 10 best-scoring structures in rank order by energy after 1000 independent simulations as well as a plot of the energies of all 1000 structures created. Each point on the plot represents a structure created by the server (Figure 7D). The x-axis is a distance from the starting position, and the y-axis is the score (energy) of the structure. A hallmark of a successful run is an energetic “funnel” of low-energy structures clustered around a single position. Lührs et al. [24] also reported that residues 1–17 of the Aβ_1–42_ are disordered; however, residues 18–42 form a beta strand–turn–beta strand motif that contains two parallel, in-register beta sheets that are formed by residues 18–26 (β1) and 31–42 (β2). Intermolecular side chain contacts are formed between the odd-numbered residues of strand β1 of the *n*th molecule and the even-numbered residues of strand β2 of the (*n* − 1)^th^ molecule. This interaction pattern leads to partially unpaired β-strands at the fibrillar ends, which explains the sequence selectivity, cooperativity, and apparent unidirectionality of Aβ fibril growth [24].

#### Binding sites/pocketness of the monomer and dimer

The binding and active sites of proteins are often associated with structural pockets and cavities. The results of analyses of pocketness of the monomer and dimer are shown in Figure 7E and G, respectively. In the A1 monomer, the cluster with the highest degree of pocketness was located between residues 17–20 and 35–42 (Figure 7F). The degree of pocketness was higher in the A1–B1 dimer (Figure 7H). The Q-site finder also identified pocketness in similar regions of the monomers and dimer (data not shown).

#### Aggregation-prone amino acid residues of Aβ_1–42_

**Figure 8:**
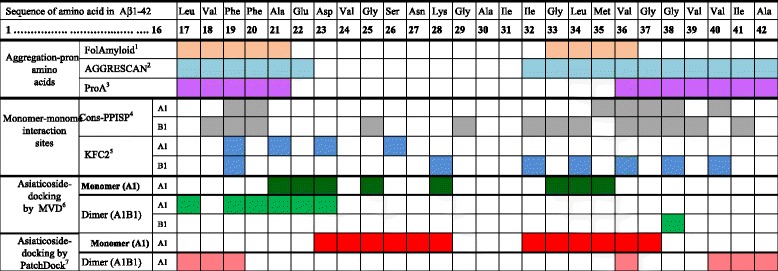
Analyses of aggregation-prone amino acid residues of Aβ1–42 peptide chain and monomer(A1)-monomer(B1) (A1B1dimer) interaction-sites, and interaction-sites between asiaticoside vs. monomer (A1) and dimer (A1B1) after docking. ^1^FoldAmyloid describes a method of prediction of amyloidogenic regions from protein sequence [27], while ^2^AGGRESCAN predicts and evaluates of “hot spots” of aggregation in polypeptides. By identifying aggregation-prone segments in proteins, AGGRESCAN facilitates the identification of possible therapeutic targets for anti-depositional strategies in conformational diseases [28]. ^3^ProA: Protein Aggregation Prediction Server, which provides access to two protein aggregation propensity prediction algorithms, based on the amino acid physicochemical properties important to protein aggregation [29]. ^4^Cons-PPISP is a consensus neural network method for predicting protein-protein interaction sites. Given the structure of a protein, cons-PPISP predicts the residues that likely form the binding site for another protein. The inputs to the neural network include position-specific sequence profiles and solvent accessibilities of each residue and its spatial neighbors. The neural network is trained on known structures of protein-protein complexes [32]. ^5^KFC2 (*Knowledge-based FADE* and *Contacts*) server predicts binding “hot spots” within protein-protein interfaces by recognizing structural features indicative of important binding contacts. The server analyzes several chemical and physical features surrounding an interface residue and predicts the classification of the residue using a model trained on prior experimental data [33,34]. ^6,7^For comparison, whether aisaticoside-binding site overlaps with the aggregation-prone hot-spot amino acids and/or the monomer-monomer (A1-B1dimer) intersurface interaction sites, the docking results, derived from the Molegro Virtual Docker (MVD)6 [23] and PatchDock7 [35] of Figure 9 are also shown here.

The hotspots for aggregation are shown in Figure 8. The FoldAmylod analysis revealed that residues 17–21 and 33–36 of Aβ_1–42_ were aggregation-prone, whereas the AGGRESCAN analysis identified residues 17–22 and 29–42 as the aggregation-prone regions. Residues 17–20 and 35–42 of Aβ_1–42_ also exhibited a propensity for aggregation upon cross-examination using the ProA server.

**Figure 9:**
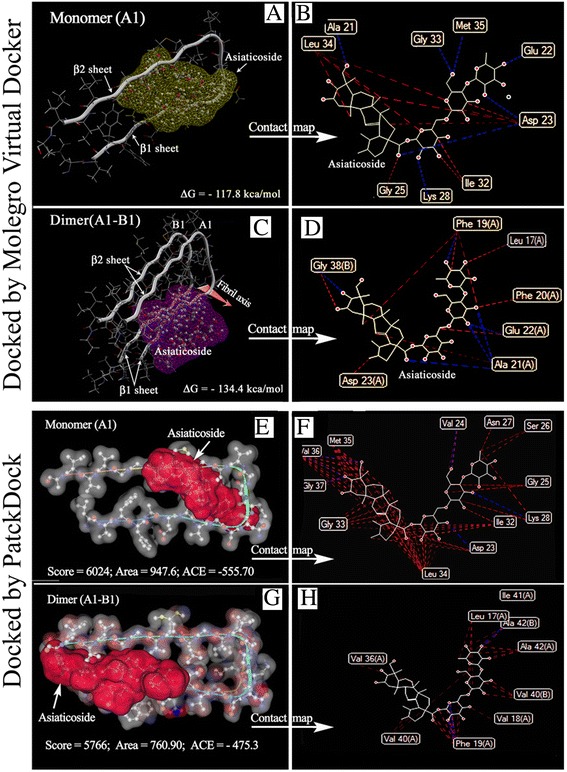
Docking poses of asiaticoside onto Aβ_1–42_ monomer and dimer. Best docking poses of the ligand (asiaticoside) and receptor (monomer and dimer) by the Molegro Virtual Docker **(A,** **B,** **C,** **D)** and PatchDock **(E,** **F,** **G,** **H)**. **A**, **C**: Snapshot of asiaticoside in the best pose 1 docked onto the A1 monomer and A1-B1 dimer. The interaction (binding) energy (−ΔG), as the MolDock score, is shown in the figures. PatchDock (E,F,G,H): Snapshot of the Patch docking of asiaticoside on to monomer **(E)** and dimer **(G)** on the geometry-based molecular docking algorithm. The best-scored complex is shown here by the Marvin molecular viewer. Score: Geometric shape complementarity score. Area: Approximate interface area of the complex (receptor-ligand), ACE: Atomic contact energy. Atomic contact maps the receptor-ligand (Aβ_1–42_-asiaticoside) complexes (B, D of Molegro docker; F, H of PatchDocker) and hydrogen maps were delineated by feeding them to Molecular Viewer. Red line indicates hydrophobic interactions, Blue line indicates hydrogen bonds.

#### Monomer–monomer and dimer intersurface interaction sites

The amino acid residues involved in intersurface interactions are also shown in Figure 8. The intersurface interaction sites of the A1–B1 dimer included Phe19, Phe20, Met35, Val36, Gly37, Gly38, and Val40 of the A1 monomer and Val18, Phe19, Phe20, Ile32, Gly33, Leu34, Val36, Gly37, Val39, and Ile41 of the B1 monomer. The intersurface analysis also revealed that amino acids Gly25, Gly29, and Gly38 of B1 monomer remained buried in the interior of the dimer. The intersurface interaction sites were also rechecked using the KFC2 server, and the sites were located on both the A1 and B1 monomers (Figure 8).

### Docking results

#### Molegro Virtual Docker (MVD)

Docking of asiaticoside with the monomers generated five poses with unique chemical arrangements. In MVD, the best pose is assessed on the basis of scoring function (MolDock score). The score (Kcal/mol) mimics the potential energy change, when the protein and ligand come together. This means that a very negative score corresponds to a strong binding and a less negative or even positive score corresponds to a weak or non-existing binding. Water molecules were not included in the docking setup, thus the ‘water-ligand interaction’ did not contribute to the MolDock score. The MolDock score of pose 1 was lower than that of the other four poses (pose 1 = −117.85, pose 2 = −117.10, pose 3 = −107.27, pose 4 = −105.83, and pose 5 = −104.92 Kcal/mol), indicating the structure in pose 1 was superior. The MolDock scores of the five poses for docking with the dimer were as follows: pose 1 = −134.48, pose 2 = −118.74, pose 3 = −118.64, pose 4 = −113.89, and pose 5 = −109.09 Kcal/mol. The results of the computational docking of asiaticoside with the A1monomer and A1-B1dimer are shown in Figure 9. Figure 9A presents the findings for pose 1 docked onto the A1 monomer, and Figure 9C presents data for pose-1 docked onto the dimer (A1-B1). The amino acids with which asiaticoside bound when it docked onto the monomer (A1) and dimer (A1–B1) were visualized by the contact maps between the atoms of asiaticoside and the Aβ_1–42_ amino acid residues using MVD (Figures 8 and 9B and 9D). Residues Ala21, Glu22, Asp23, Gly25, Lys28, Gly33, Leu34, and Met35 of the A1 monomer bound with asiaticoside (Figure 9B). The amino acid residues of the A1–B1 dimer that sterically interacted with asiaticoside were Leu17, Phe19, Phe20, Ala21, Glu22, Asp23 (from the A1 monomer), and Gly38 (from the B1 monomer) (Figure 9D). The higher free energy of binding between asiaticoside bound to the dimer than that of asiaticoside bound to the monomer suggests a stronger binding with the dimer.

#### PatchDocker

The PatchDock server provided a web page that presents the top 20 solutions. The best-scored receptor-ligand complex (Top 1) was visualized by Marvin Space (Figure 9E, 9G). Afterwards, the Top1 complex was subjected to Molegro Virtual Viewer (MVV) to show the atomic contact maps between receptor-ligand complex (Figure 9F, 9H). The results of the PatchDock revealed that amino acids Asp23,Val24, Gly25, Ser26, Asn27, Lys28, Ile32, Gly333, Leu34, Met35, Val36 and Gly37 of the monomer bound (contacted) with asiaticoside (Figure 9F). While Aβ_1–42_ dimer was docked with asiaticoside, the amino acid residues Leu17, Val18, Phe19, Val36, Val40, Ile41 and Ala42 of A1-monomer, and those of the Val40 and Ala42 of B1-monomer bound with asiaticoside (Figure 9H). The common features between Molegro Virtual Docker and PatchDocker were displayed in their common amino acid binding sites for asiaticoside: Asp23, Gly 25, Lys28, Gly33, Leu34 and Met35 of monomer were bound with asiaticoside in both of the docking systems. Consistently, the amino acids Leu17 and Phe19 of A1 monomer were common when the dimer (A1-B1) was docked with asiaticoside by both the Molegro Virtual Docker and PatchDocker.

## Discussion

Fluorescence correlation spectroscopy (FCS) is a method based on fluorescence intensity fluctuations that result from dynamic movement and fluorescence quantum yield of fluorescent molecule while it diffuses in and out of small detection area. In this study we recorded the effect of asiaticoside on the dynamical properties of Aβ_1–42_ fibrillation in FCS. The initial steps of Aβ aggregation are preceded by the transformation of α-sheets to β-sheets, through lateral stacking of dimers, trimers, tetramers, and oligomers, leading to matured amyloid fibers [21]. Autocorrelation function of FCS analysis revealed a diffusion time of 100 ± 5 μs for the reference dye TAMRA, vs. 150 ± 5 μs for TAMRA-Aβ_1–42_ alone. The increased diffusion time of the latter is consistent with its increased molecular mass. At time zero, the diffusion time of TAMRA-Aβ_1–42_ was not significantly different in the presence or absence of asiaticoside, demonstrating that the amyloids were in their monomeric state at the start and/or early steps of fibrillation. After 1 h of incubation, the diffusion time of TAMRA-Aβ_1–42_ in the TAMRA-Aβ_1–42 +_ unlabeled Aβ_1–42_ samples increased to 208 ± 4 μs, indicating that amyloid species/units with a molecular mass relatively greater than the previous one occurred in the early steps, including oligomrizations of Aβ fibrillation process. This resulted in a concurrent slowness of movements of the amyloid units during “entering-and-leaving” the confocal volume of FCS. In FCS, larger amyloid aggregates (of Aβ_1–42_) in solution diffuse less frequently through the confocal volume, thereby resulting in longer diffusion times. Thus, the decreased diffusion time in the presence of asiaticoside reflects an inhibition of Aβ_1–42_ aggregation by this glycoside, allowing more Aβ_1–42_ in the confocal volume to freely diffuse in and out of the confocal volume. Repeated FCS measurements of 10-s sampling times illustrated an increase in the amplitude (y-axis). The amplitude reflects the inverse of the average number of fluorescent TAMRA-Aβ_1–42_ molecules in the focal volume. In other words, the correlation amplitude increases in size as the intensity fluctuation grows larger. This again demonstrates that asiaticoside inhibited amyloid fibrillation, allowing an increasing number of free TAMRA-Aβ_1–42_ molecules to freely diffuse in the confocal volume. The diffusion time decreased as the asiaticoside concentration increased (Figure 3C).

Recently, *C. asiatica* extract, which contains two major triterpene glycosides (asiaticoside and madecassoside) as active components, is increasingly being used to enhance memory [9], which is severely impaired in patients with AD. Very recently, we have reported that the oral administration of madecassoside improves the memory loss of AD model rats, with concurrent inhibition of Aβ_1–42_ fibrillation [36]. In the present study, asiaticoside inhibited Aβ_1–42_ fibrillation at the early stages of the process, hinting at the basis of the neuroprotective effects of *C. asiatica* extract and/or its active components (asiaticoside and/or madecassoside) against Aβ_1–42_–induced AD.

Steady-state ThT fluorospectroscopy also revealed that asiaticoside displayed the strongest inhibition of fibrillation at 20 μM. The results evolved from steady-state ThT fluorospectroscopy, however, did not directly disclose about the stage(s) at which asiaticoside exerted the inhibitory effect on fibrillation. Therefore, we evaluated the effect of asiaticoside on the kinetics of Aβ_1–42_ fibrillation. The ThT growth curve for Aβ_1–42_ was sigmoidal showing a dependence on time, which suggests that the amyloid assembly process is cooperative. The presence of asiaticoside led to a significant increase in the lag phase of Aβ_1–42_ fibrillation with a concurrent deceleration of the exponential growth of fibrils. Thus it would appear that the asiaticoside acted to reduce the rate of nucleation, and, however, once critical nuclei were formed elongation was also affected by the presence of the asiaticoside. Once the FCS measurements and kinetic and/or steady-state ThT-fluorospectroscopic measurements proved that asiaticoside delayed/inhibited fibrillation, we also examined the effect of asiaticoside on amyloid fibrillation using Laser Scanning Microscopy (LSM). The ThT-bound amyloidal aggregates (green) were clearly visible in the confocal images. The red fluorescence areas indicate the larger aggregates of TAMRA-Aβ_1–42_-intercalated fibers of Aβ_1–42_ (5 nM TAMRA-Aβ_1–42_ was used in excess of unlabeled Aβ_1–42_). Total numbers of fluorescent aggregates of Aβ_1–42_ control samples were much more frequent and larger than those of the Aβ_1–42_ + asiaticoside samples (Figure 5). Therefore, the LSM results are consistent with those of the FCS and steady-state/kinetics of ThT fluorospectroscopic studies, illustrating that asiaticoside exhibits a strong anti-fibrillation effect.

We directly observed the high-resolution image of the dense aggregates of matured fibers, long/short or single/few scattered matured fibrils, typical branched fibrils, protofibrils, oligomer-like, in some instances, amorphous and other unstructured smaller aggregates of Aβ_1–42_ in the grid fields of TEM (Figure 6). This suggests that fibrillation of Aβ_1–42_ goes through a diverse dynamic exchange of conformational stages and includes continuous shuffling and growth, particularly, during the first stages of aggregation; Aβ_1–42_ can adopt, at least, a globular structure or so-called nucleating center. These globular structures form oligomers leading to protofibrils, which then associate in elongated fibers, finally to the highest ordered matured Aβ_1–42_ fibers. Asiaticoside-induced interference of Aβ_1–42_ fibrillation also was evident from the morphology of Aβ_1–42_ fibrils in transmission electron micrographs. Consistent with the fact that pretreatment of Aβ_1–42_ with asiaticoside kinetically lengthened the formation of amyloid nucleating centers and reduced the protofibrillar structures, TEM grids of the Aβ_1–42_ + asiaticoside samples were also devoid of complete fibers, and only a small number of fibrils exhibited a diffused, shorter and some spheroid-type morphology. Structural characterization of these spheroidal species has remained elusive. Soluble amyloid β-oligomeric intermediates, emerging during amyloid fibril assembly, represent the main molecular species responsible for toxicity to cells and tissues [15]. The presence of these indefinable spheroidal amyloid species in TEM thus may raise the question of whether aisiticoside would confer toxicity to cells, and deteriorate Alzheimer’s symptoms. However, there are reports that proteins can assemble into ordered aggregates without invoking the cross-β motif [37]. Ehrnhoefer et al. [38] showed that the polyphenol epigallocatechine gallate redirected Aβ_1–42_ into unstructured and nontoxic off-pathway oligomers. Asiaticoside-induced decreases in the formation and quantity of amyloid fibers (Figures 4, 5 and 6) are thus qualitatively consistent with these reports. Therefore we speculate that the spheroidal species of amyloids seen in the TEMs of asiaticoside + Aβ_1–42_ samples are not toxic oligomer species. Otherwise, asiaticoside containing *centella asistica* extract couldn’t have beneficial effects on memory [9], dementia and cognition [11], neuronal damage [39], neurite outgrowth [40] and Aβ_1–42_-induced neurotoxicity in AD model animals [10,41]. Our previous results with madecassoside are also consistent with the speculation [36]. Because asiaticoside kinetically decreased the diffusion time, and inhibited ThT-determined lag and growth phase of fibrillogenesis, the morphological transformation of Aβ_1–42_ may be mediated by the effects of this glycoside.

Structure-based drug design has made tremendous contributions to the fields of cancer chemotherapy and drug-resistant infections. Computational structure-based drug design may, therefore, facilitate the development of novel treatments for AD. Therefore, the docking of asiaticoside with the Aβ_1–42_ monomers and dimer was used in combination with experimental data to obtain information on the binding domain and mechanism of inhibition. The ability of Aβ_1–42_ to bind to itself and to different ligands in a highly specific manner is an important feature of amyloidogenesis. Kirschner et al. [42] reported that β-sheets run parallel and β-strands run perpendicular to the fibril axis. Sheet β1 is formed by approximately residues 18–26, and sheet β2 is formed by approximately residues 31–42. Thus, the corresponding residues of adjacent monomers line up along the fibril [24]. The aggregation-prone amino acids, i.e., hotspots of aggregation of Aβ_1–42_, were located in the regions of residues 17–22 and 32–42 Figure 8, which matched to the β1 and β2 sheets of 2BEG and interaction sites of asiaticoside, respectively. While docked with the monomer in MVD (Figure 9A), asiaticoside displayed steric interactions with Ala21, Glu22, Asp23, and Gly25 of the β1 sheet and Lys28, Gly33, Leu34, and Met35 of the β2 sheet, thereby indicating that the formation of β-sheets (from the native α-helix) may have been, at least partially, perturbed by asiaticoside. PatchDocking of asiaticoside onto Aβ_1–42_ also revealed that asiaticoside bound with the amino acid sites located at the β1 and β2 domain of the Aβ_1–42_ (Figure 9E-H). Remarkably, both the β1 (residues 17–21) and β2 (residues 31–42) sheet-forming amino acids were engaged in monomer–monomer (dimer) intersurface interactions (Figure 8). These results, therefore, suggest that the intersurface interaction sites could be used as the ultimate target sites of ligands to inhibit the interactions. We previously reported that the dimer may act as one of the seeding units of Aβ_1–42_ protofibrils, leading to matured fibers [21]. This stimulated us to dock the dimer (Figure 9C, G) with asiaticoside to clarify whether the docking affects the intersurface interaction sites. As expected, asiaticoside bound with the amino acids of these sensitive regions. These results again suggest that asiaticoside impedes both the β-sheet-forming amino acid residues and binds with the amino acids that remain located in the monomer–monomer intersurface and buried in the loop region, such as Gly38 (Figure 8).

## Conclusions

Studies of aggregation of Aβ_1–42_ and interactions between small molecules, such as, asiaticoside and Aβ_1–42_ may contribute to the development of inhibitors against Alzheimer’s disease. In this study, by means of a combined approach of ThT, FCS, LSM, TEM techniques, we suggest a model of how the asiaticoside interacts with β_1–42_. Our experimental results allow us to conclude that asiaticoside inhibits early stages of fibrillogenesis through interactions with ‘nucleating’ amyloid species and decelerating the growth phase. In addition, the *in silico*, docking of asiaticoside with Aβ_1–42_ appears to be consistent with the ability of this glycoside to inhibit fibrillation by acting both at the intra- and inter- β-sheet interaction sites. Finally, the results obtained support further development of asiaticoside for clinical use in Aβ_1–42_ -induced neurodegenerative diseases, such as Alzheimer’s disease.
